# Reduction of hRNase H2 activity in Aicardi–Goutières syndrome cells leads to replication stress and genome instability

**DOI:** 10.1093/hmg/ddu485

**Published:** 2014-09-30

**Authors:** Sara Pizzi, Sarah Sertic, Simona Orcesi, Cristina Cereda, Marika Bianchi, Andrew P. Jackson, Federico Lazzaro, Paolo Plevani, Marco Muzi-Falconi

**Affiliations:** 1 Dipartimento di Bioscienze, Università degli Studi di Milano, 20133 Milano, Italy; 2 Child Neurology and Psychiatry Unit; 3 Laboratory of Experimental Neurobiology, C. Mondino National Neurological Institute, Pavia, Italy and; 4 Medical Research Council Human Genetics Unit, MRC Institute of Genetics and Molecular Medicine, University of Edinburgh, Edinburgh EH4 2XU, UK

## Abstract

Aicardi–Goutières syndrome (AGS) is an inflammatory encephalopathy caused by defective nucleic acids metabolism. Over 50% of AGS mutations affect RNase H2 the only enzyme able to remove single ribonucleotidemonophosphates (rNMPs) embedded in DNA. Ribonucleotide triphosphates (rNTPs) are incorporated into genomic DNA with relatively high frequency during normal replication making DNA more susceptible to strand breakage and mutations. Here we demonstrate that human cells depleted of RNase H2 show impaired cell cycle progression associated with chronic activation of post-replication repair (PRR) and genome instability. We identify a similar phenotype in cells derived from AGS patients, which indeed accumulate rNMPs in genomic DNA and exhibit markers of constitutive PRR and checkpoint activation. Our data indicate that in human cells RNase H2 plays a crucial role in correcting rNMPs misincorporation, preventing DNA damage. Such protective function is compromised in AGS patients and may be linked to unscheduled immune responses. These findings may be relevant to shed further light on the mechanisms involved in AGS pathogenesis.

## INTRODUCTION

Aicardi–Goutières syndrome (AGS) is a rare and underdiagnosed inflammatory encephalopathy with infancy onset and characterized by high levels of Type I interferon (IFN) production. AGS is caused by defective nucleic acids metabolism due to alterations in different nucleases or nucleotidases (1–4). The majority of AGS patients carry mutations in one of three genes coding for RNase H2 subunits (RNASEH2A, RNASEH2B, RNASE2HC, also classified as AGS4-2-3, respectively).

RNases H are specialized enzymes that process the RNA moiety in RNA : DNA hybrid molecules. These hybrid structures represent physiological intermediates produced during retroviral infection, retroelement mobilization and during genome replication, through the synthesis of Okazaki fragments or when a replication fork collides with the transcriptional machinery (5,6). Two classes of RNases H, with partially overlapping substrate specificity, have been characterized (7). RNase H1 requires a stretch of at least four consecutive ribonucleotidemonophosphates (rNMPs) to cleave; in mammals RNase H1 is essential for mitochondrial DNA replication and the function of the nuclear form is still unclear (8,9). RNase H2 is a trimeric complex that, besides being able to process long RNA : DNA hybrid molecules, has the unique property of cleaving single rNMPs embedded in genomic DNA. A new and potentially relevant substrate for RNase H2 has been recently identified. Indeed, recent evidence revealed that ribononucleotide triphosphates (rNTPs) are misincorporated into genomic DNA with high frequency during normal replication (10–12). Due to the reactive 2′ hydroxyl group in the ribose moiety, RNA is ∼100 000-fold more susceptible than DNA to spontaneous hydrolysis under physiological conditions (13). The choice of DNA instead of RNA as the information storage molecule is critical for genome stability. Stable incorporation of rNTPs in DNA needs to be avoided, as it makes DNA prone to strand breakage and mutagenesis (14–16). DNA polymerases have evolved active sites that distinguish between rNTPs and deoxyribonucleotide triphosphates (dNTPs), and select the latter for DNA replication (17). However, the fidelity of DNA polymerases is challenged by the high ratio of rNTPs to dNTPs that ranges from 10- to 100-fold in *Saccharomyces* *cerevisiae* (10) and in mammalian cells (18). Moreover, rNTPs may be added to DNA filaments during repair of double-strand breaks (DSBs) in G1 (19,20) and frequent rNTPs incorporation was observed during HIV-1 reverse transcription (21). Altogether, these findings established that incorporation of rNTPs in genomic DNA is the most frequent source of endogenous DNA modification in replicating cells, and it is well established that cells have evolved various surveillance mechanisms to preserve genome integrity during DNA replication and facilitate repair (22–24).

Budding yeast cells carrying combined deletions of RNase H1 and RNase H2 genes are viable, although they show evident cell growth defects due, at least partly, to the accumulation of genomic rNMPs (25). Conversely, both RNase H1 and RNase H2 null mice die during embryogenesis, demonstrating the essential function of these enzymes in mouse development (9,11,12). Concordantly, only hypomorphic RNase H2 mutations have been reported in AGS patients, suggesting an essential role for RNase H2 (2,26–29). In vertebrates, studies investigating the effect of RNase H2 dysfunction have been carried out in mouse embryonic fibroblasts (11,12). Studies in human cells, modulating the expression of the RNase H2 genes by RNA interference and exploiting patients-derived cell lines, would be useful to identify the molecular mechanisms perturbed by RNase H2 defects in AGS.

To characterize the effects of RNAse H2 dysfunction, we used both ***AGS2, AGS4-mutated cells and lentiviral vectors carrying specific shRNA sequences to induce stable RNase H2 knockdown in human cell lines. Here, we report that depletion of RNase H2 in culture cells or AGS hypomorphic mutations in patients-derived lymphoblastoid cells lead to the accumulation of genomic rNMPs, causing endogenous replication stress, as evidenced by impaired cell cycle progression and chronic post-replication repair (PRR) activation, and trigger the DNA damage response (DDR). The gravity of the phenotype correlates with the silencing efficiency or the severity of the mutation. Intriguingly, recent studies linked DDR to activation of an immune response, suggesting a possible mechanism for the pathogenesis of AGS linked to defective RNase H2 functions.

## RESULTS

### RNase H2 depletion in human cells impairs normal cell proliferation

Recent *in vivo* studies in yeast and mouse cells suggest a role for RNase H2 in the maintenance of genome stability through the removal from genomic DNA of rNMPs misincorporated during the replication process (11,12,25). However, the link between this role of RNase H2 and the pathogenesis of AGS is unknown.

To better understand the consequences of reduced RNase H2 activities in human cells, we used HeLa cells where the level of RNase H2 can be modulated by RNA interference. To achieve a stable down-regulation of RNase H2, we generated a variety of lentiviral vectors carrying shRNA sequences specifically designed to interfere with the expression of either the catalytic A subunit or the structural B subunit, which are connected, respectively, to the most severe phenotype or the most common mutations found in AGS patients (30). The degree of silencing was evaluated using quantitative reverse transcriptase-polymerase chain reaction and/or western blotting using commercial antibodies or antibodies developed in-house (Supplementary Material, Fig. S1).

**Figure 1.**
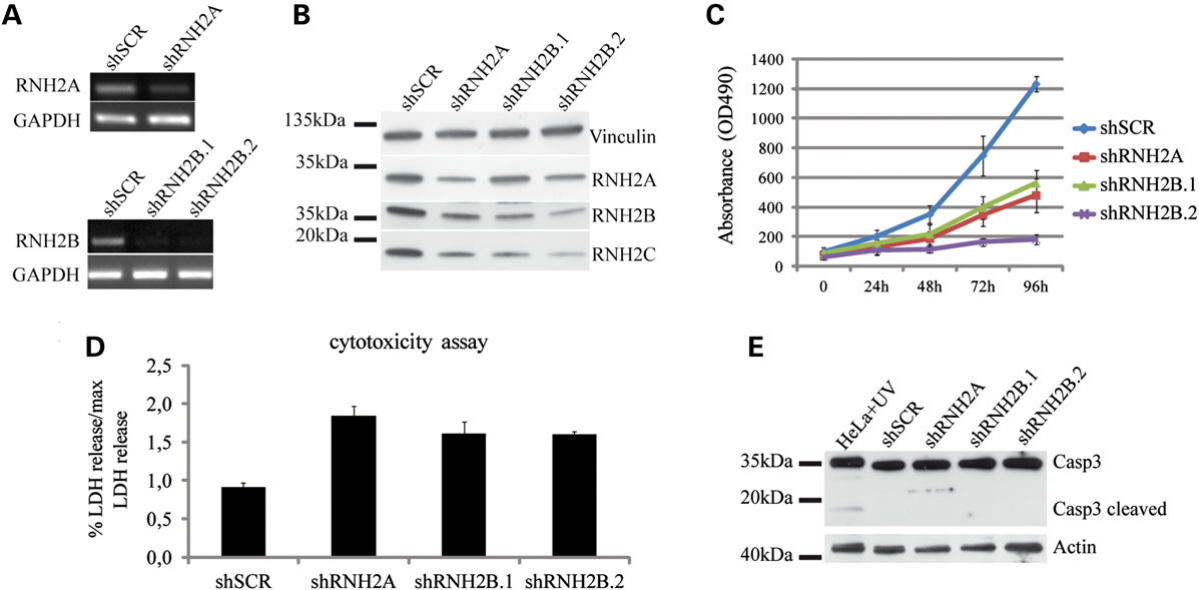
Low levels of RNase H2 reduce cell proliferation. HeLa cells were silenced using one shRNA sequence against subunit A and two different sequences against subunit B (shRNH2A, shRNH2B.1, shRNH2B.2). As control, non-targeting shSCRAMBLE (shSCR) sequence was used. Efficiency of silencing was monitored by RT-PCR (**A**) or immunoblotting (**B**). Silencing of A or B subunits affects protein level of subunit C (B). MTS assay was performed in triplicate to monitor cell proliferation. Cells were plated 7 days after infection and analyzed every 24 h. Mean ± s.e.m. (**C**). Relative LDH release was measured 11 days after infection. Mean ± s.e.m. of triplicate (**D**). Representative graphs are shown to avoid blending of the data due to variation in timing and efficiency of silencing. All experiments were performed three times and produced similar results. Immunoblot for Caspase3 was performed on total protein extract 11 days after infection. Protein extract from HeLa cells treated with H_2_O_2_ were used as positive control (**E**).

After lentiviral infection, HeLa cells were analyzed for silencing efficiency (Fig. 1A and B). As shown in Figure 1B, western blot analysis revealed that down-regulation of any RNase H2 subunit affects the expression levels of the other subunits, likely due to destabilization of the entire heterotrimeric complex. Upon depletion of RNase H2, proliferation of HeLa and MRC5VI cells was significantly reduced compared with control cells (Fig. 1C; Supplementary Material, Fig. S2) and this effect seems to increase with the efficiency of silencing (Supplementary Material, Fig. S3). The proliferation defect can be only partially ascribed to cell death, as revealed by the modest increase in lactate dehydrogenase (LDH) release respect to control cells; furthermore, silenced cells did not exhibit evident Casp3 cleavage, a marker of Casp3-mediated apoptotic pathway activation (Fig. 1D and E).

These findings suggest that in human cells a decreased level of RNase H2 affects cell proliferation through mechanisms that cannot be compensated by RNase H1 activity.

### Depletion of RNase H2 causes replication stress and chronic activation of PRR

Given that RNase H2 silencing had only a limited impact on cell vitality, we investigated the possibility that loss of RNase H2 function may lead to defective cell cycle progression.

**Figure 2.**
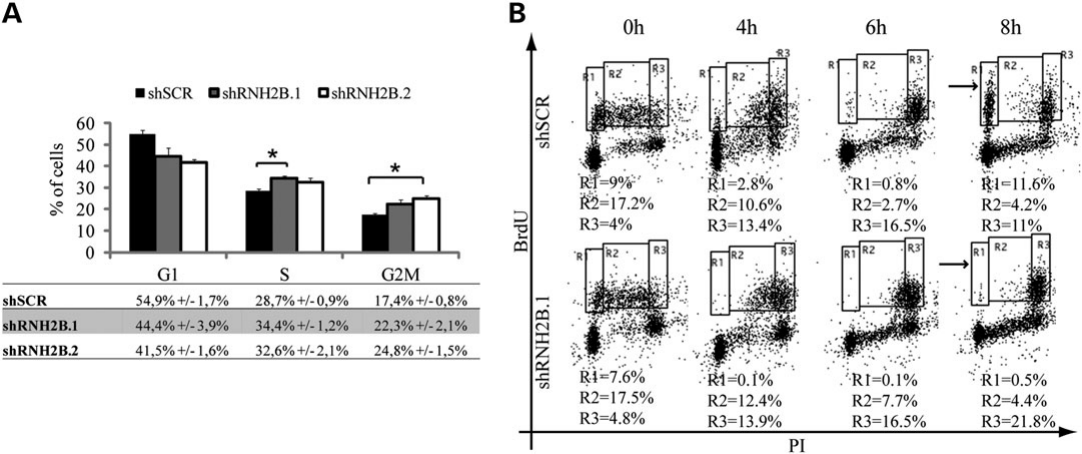
RNase H2 depletion causes a delay in S-G2 phases. Cell cycle distribution of asynchronous HeLa cells. Histograms represent the percentage of cells in G1, S and G2-M phase. FACS profiles were analyzed using CellQuest software. Data are mean ± s.e.m. of four independent experiments. *P < 0.02 (*t*-test) (**A**). Representative images of a cell kinetic experiment. Asynchronous HeLa cells were pulse labeled with BrdU and released in BrdU-free medium. Cells were harvested and stained with anti-BrdU and propidium iodide (PI) at indicated time points (hours after release). R1 = early S-phase; R2 = mid-S-phase; R3 = late S/G2 phase (arrows indicate cells entered in new G1 phase) (**B**).

Exponentially growing HeLa cells were labeled with BrdU to mark replicating cells, and analyzed by cytofluorimetry for total DNA content and for active replication. RNase H2 silencing affected cell cycle progression, resulting in a reproducible and significant accumulation of cells in S and G2-M phases (Fig. 2A). Cell cycle progression was further analyzed in a kinetic experiment where cells traversing S-phase were labeled with a pulse of BrdU and followed for 8 h after release in BrdU-free medium. When compared with the SCRAMBLE control, cells depleted for RNase H2 were delayed in late-S/G2 phase and were very slow in getting back into G1 phase (Fig. 2B), indicative of replication problems. Similar results were obtained using MRC5VI cells, indicating that it is not a cell-specific effect (Supplementary Material, Fig. S4A).

Our previous work in budding yeast demonstrated that, in the absence of RNase H activity, misincorporated rNMPs challenge the efficiency of the replication machinery and *rnh1Δ rnh201Δ* cells can cope with such problem by activating the PRR repair pathway to bypass the blocking ribonucleotide (25). We hypothesized that a similar problem may be responsible for the defects in completing S-phase and for the temporary G2/M arrest observed in HeLa cells after RNase H2 down-regulation. To evaluate the involvement of PRR, we analyzed the level of ubiquitylated proliferating cell nuclear antigen (PCNA), a biochemical readout of PRR activation, in RNase H2-silenced cells.

**Figure 3.**
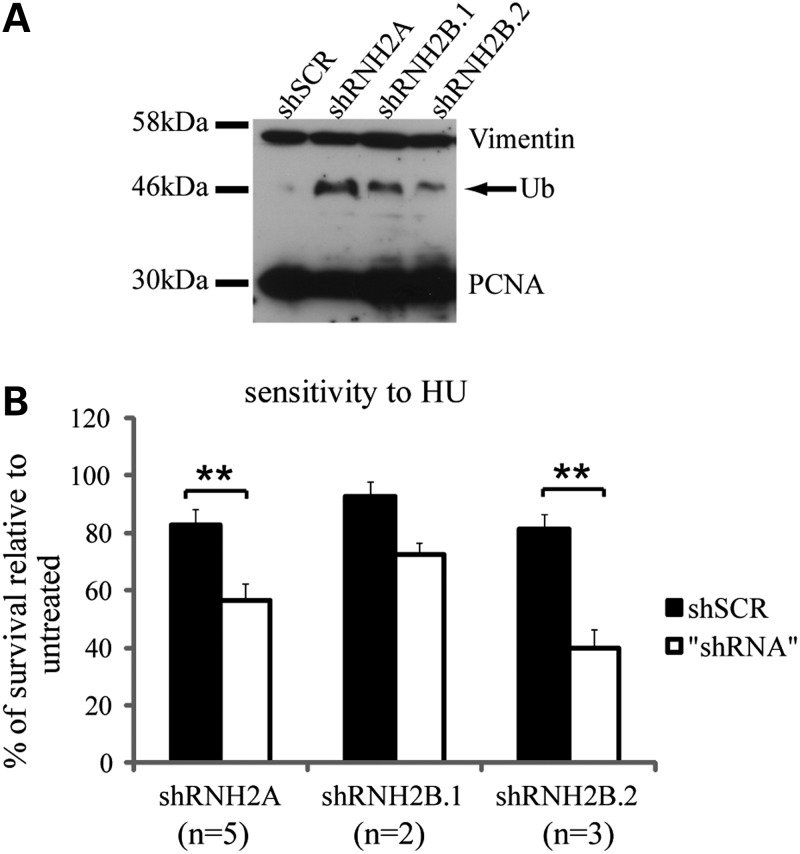
Inactivation of RNase H2 induces replication stress. Immunoblot on total protein extract from control and RNase H2A/B silenced HeLa cells against PCNA. Vimentin was used as loading control (**A**). Sensitivity to HU was evaluated by colony forming assay. Control and silenced HeLa cells were incubated for 14 days with or without HU 0.1 mm. The histograms report the percentage of surviving colonies respect to the untreated sample. Experiments were performed in duplicate or triplicate. Error bars represent mean ± s.e.m. (**B**). **P < 0.003 (*t*-test).

Western blotting analysis on protein extracts obtained from exponentially growing cells, revealed an accumulation of ubiquitylated PCNA in cells depleted of RNase H2A or RNase H2B, indicating that if RNase H2 is not fully functional, cells use the post-replication repair pathway to complete replication (31) (Fig. 3A). This hypothesis was strengthened by the observation that RNase H2-depleted cells are hypersensitive to low doses of the replication stress-inducing agent hydroxyurea (HU) (Fig. 3B; Supplementary Material, Fig. S4B). Low levels of HU decrease the levels of dNTPs pools and are toxic for cells suffering endogenous replication stress, while they are well tolerated by cells normally traversing replication phase (25,32,33) supporting the notion that RNase H2 depletion impairs normal replication fork progression.

### Depletion of RNase H2 triggers the activation of genome integrity maintenance pathways in human cells

Given the problems in completing chromosomal DNA replication and the delay in traversing mitosis into the next G1, we analyzed several markers of activated DDR pathways.

**Figure 4.**
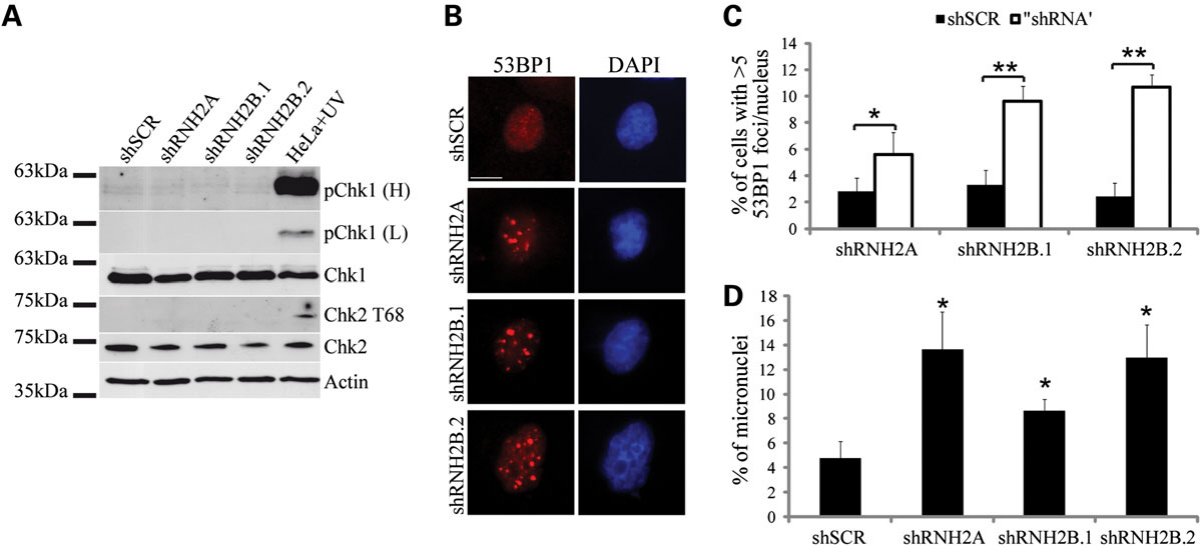
Depletion of RNase H2 induces DDR and causes genome instability. Total protein extract of silenced HeLa cells was blotted with indicated antibodies, HeLa irradiated with UV (20 J/m^2^) were used as control (pChk1, anti-Chk1 Ser317; L, low exposure; H, high exposure) (**A**). HeLa cells were immunostained with an anti-53BP1 antibody and DAPI to stain nuclear DNA (scale bar: 10 μm, magnification ×63) (**B**). The histograms indicate the percentage of cells that have at least five 53BP1 foci/nucleus. Error bars represent mean ± s.e.m.; *n* = 4 for shRNH2A and shRNH2B.1, *n* = 5 for shRNH2B.2; *P < 0.05, **P < 0.01 (*t*-test). (**C**) The histograms in (**D**) indicate the percentage of micronucleated cells. Error bars represent mean ± s.e.m.; *n* = 4; *P < 0.02 (*t*-test).

We failed to detect damage-specific phosphorylation of Chk1 and Chk2 checkpoint kinases (Fig. 4A). On the other hand, RNase H2 silenced cells, analyzed at single cell level by immunofluorescence, exhibit a clear increase in 53BP1 foci formation (Fig. 4B and C; Supplementary Material, Fig. S5A and B). 53BP1 is a well-known mediator of DNA DSB repair, and its accumulation in foci has been recently associated to replication stress (34–37). Furthermore, depletion of RNase H2A or RNase H2B leads to the formation of micronuclei (Fig. 4D; Supplementary Material, Fig. S6), which is indicative of chromosome breakage and increased genome instability (38).

Taken together, our data reveal that human RNase H2 is required for the maintenance of genome integrity and suggest that, in its absence, genome instability may be a consequence of replication fork stalling.

### Cells derived from AGS patients exhibit chronic activation of PRR DNA damage markers

Seven genes have been found to be involved in AGS (1–4,39), but over 50% of the AGS patients carry mutations in the three genes encoding the RNase H2 subunits (3,4,30). The most frequent mutations found in patients occur in the gene encoding the structural B subunit, and are often associated to milder phenotypes. Conversely, a G37S mutation affecting the catalytic A subunit is less frequent and it is associated to severe AGS symptoms. In any case, all mutations in the three RNase H2 genes trigger, to a different extent, an increased instability of the RNase H2 enzymatic complex (2,11,26–28,40).

We wondered whether the effects we have seen in HeLa cells after down-regulating RNase H2 may have some relationship with the pathogenesis of AGS. To start approaching this question, we characterized in detail EBV-immortalized lymphoblastoid cells derived from AGS patients. In particular, we analyzed: (i) the level of the RNase H2 complex subunits, (ii) the status of post-replication repair activation and (iii) the presence of markers of DDR activation in patient-derived cells, carrying the G37S mutation in the A subunit or the A177T and the A177T-T163I hetero-compound mutations in the B subunit.

**Figure 5.**
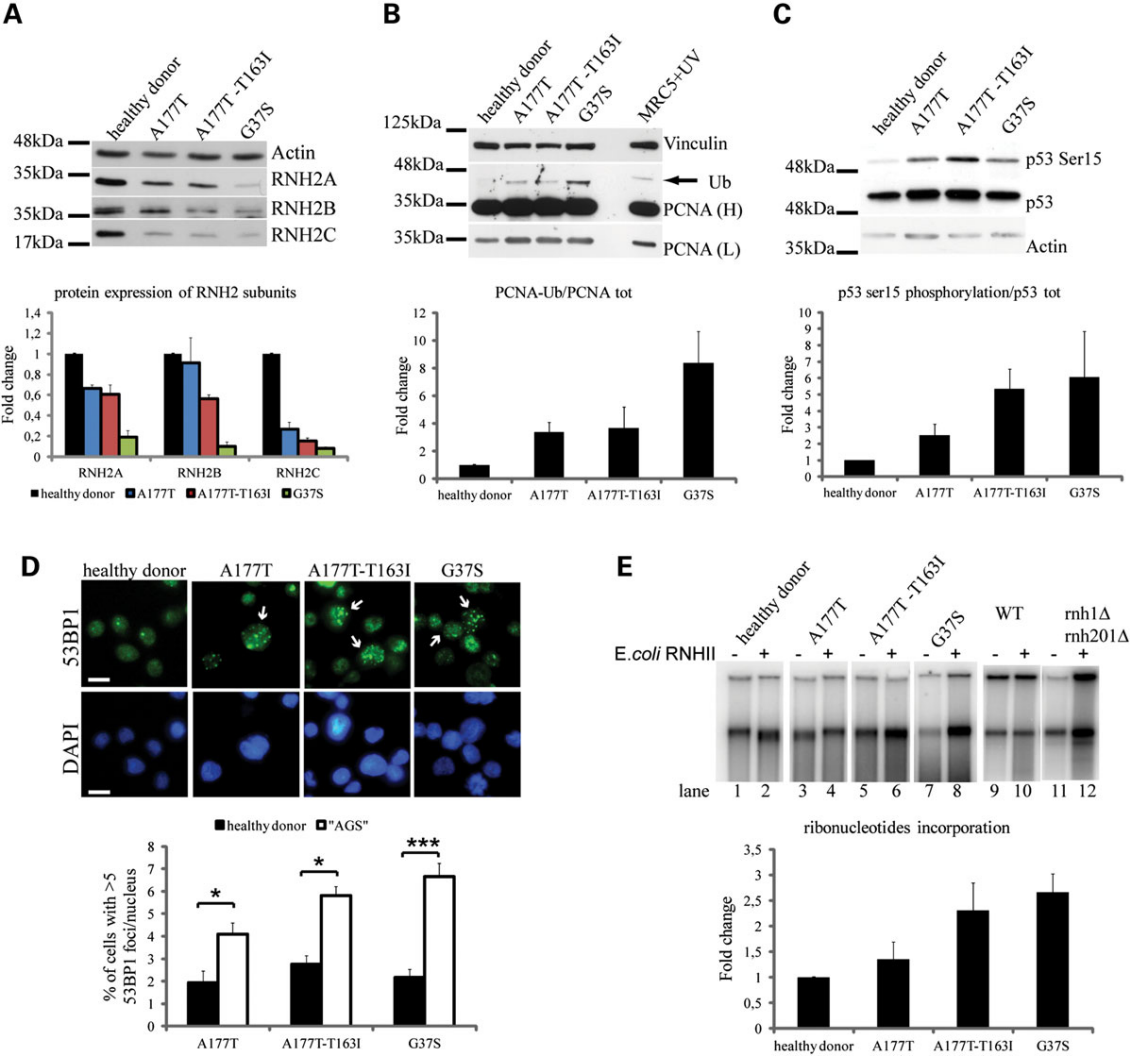
Patients-derived AGS cells accumulate genomic rNMPs and replication stress markers. Protein levels of the three subunits of RNase H2 complex from cells derived from healthy donor and AGS patients are compared by western blot (top panel) and quantified (bottom panel). Error bars represent mean ± s.e.m., *n* = 3 (**A**). Immunoblot against PCNA on total protein extract from healthy donor and AGS-derived lymphoblastoid cells, MRC5VI cells UV irradiated (20 J/m^2^) were used as positive control for PCNA ubiquitylation (L, low exposure; H, high exposure) and Vinculin as loading control (top panel). The ratio of monoubiquitinated PCNA to total PCNA is plotted (bottom panel). Error bars represent mean ± s.e.m., *n* = 5 for healthy donor, A177T, A177T-T163I; *n* = 2 for G37S (**B**). DDR in control and AGS-mutated cells was monitored by western blot analysis using anti-P-Ser-15-p53 antibody and Actin as loading control (top panel). The ratio between P-Ser-15-p53 and total p53 was quantified (bottom panel). Error bars represent mean ± s.e.m., *n* = 3 (**C**). Representative images of immunostaining anti-53BP1 and nuclei (scale bar: 10 μm, magnification ×63) (top panel). Quantification of 53BP1 foci formation in lymphoblastoid cells from healthy donor and AGS patients. Error bars represent mean ± s.e.m., *n* = 5 for A177T and A177T-T163I, *n* = 8 for G37S (bottom panel). *P < 0.02; ***P < 0.0001(*t*-test) (**D**). DNA extracted from control and AGS-mutated cells was digested (even lanes) with bacterial RNase HII before nick translation reaction in the presence of ^32^P-dCTP. Labeled DNA was run on a 1% agarose gel and visualized by autoradiography. Control samples were not digested with RNase HII (odd lanes) to detect background signal; wild-type or RNase H1 and H2 deleted (*rnh1Δrnh201Δ*) budding yeast strains were used to validate the protocol (lanes 9–12) (top panel). Graph represents quantification of three independent experiments, as described in Materials and Methods (bottom panel). Error bars represent mean ± s.e.m., *n* = 3 (**E**).

As shown in Figure 5A, both the mutation in subunit A and the mutations in subunit B have a destabilizing effect on the RNase H2 proteins. Indeed, the protein level of each RNase H2 subunit is noticeably decreased in AGS patients-derived cells, consistently with *in vitro* thermal stability assays (29). Intriguingly, the analysis of PCNA revealed clear constitutive mono-ubiquitylation in G37S-mutant cells, and a low but reproducible ub-PCNA signal in A177T and A177T-T163I cells (Fig. 5B), paralleling the severity of the phenotype reported in patients. Interestingly, phosphorylation of p53 on serine 15, an established marker of DNA damage (41), was detected at a comparable level in all mutated cells, suggesting that the impairment of RNase H2 function in patients triggers the DDR (Fig. 5C). This is further supported by the observation that 53BP1 foci are increased in all AGS-mutated cells (Fig. 5D; Supplementary Material, Fig. S7).

### Impaired RNase H2 function leads to accumulation of rNMPs in chromosomal DNA

Given the phenotypes reported above, we hypothesized that RNase H2-defective cells derived from AGS patients may accumulate rNMPs within chromosomal DNA, similarly to what was observed in mouse (11,12). To test this hypothesis, we evaluated the presence of ribonucleotides in genomic DNA preparations that were digested with bacterial RNaseHII. Nicks generated by the enzyme were then detected through the incorporation of radiolabeled dNTPs by DNA polymerase I (12). Genomic DNA from AGS and control lymphoblastoid cells was extracted and analyzed as described above. Despite a noticeable background, likely due to nicks introduced during DNA extraction, Figure 5E shows that G37S-mutated and A177T-T163I hetero-compound cells exhibit a strong rNMP-dependent radioactive signal (compare lanes 5–6 and lanes 7–8), while in A177T cells we could not detect a clear increase in the signal after treating genomic DNA with bacterial RNHII. The protocol was validated using wild-type (lanes 9–10) or RNase H1 and H2 deleted budding yeast strains (lanes 11–12). These results indicate that in cells derived from AGS patients, RNase H2 mutations affecting the catalytic activity or the stability of the complex cause the accumulation of rNMPs in genomic DNA that represent obstacles for replication forks progression, leading to fork stalling and genome instability.

## DISCUSSION

AGS, a rare genetic autoimmune disorder (30), is caused by defects in nucleic acids metabolic enzymes. Over 50% of the patients carry mutations affecting RNase H2, the main enzyme involved in the processing of single rNMPs embedded in the eukaryotic chromosomes (7,42).

Ribonucleotides recently emerged as the main non-canonical nucleotides present in genomic DNA (14,16); indeed, they are incorporated into DNA by replicative polymerases with surprisingly high frequency (∼1rNMP/1000dNMP) (10). Moreover, it is possible that imperfect processing of Okazaki fragments contribute to increase the load of genomic rNMPs. Although the presence of rNMPs within chromosomal DNA has been shown to have relevant physiological functions, acting as imprint signals and directing the mismatch repair machinery to correct the newly synthesized DNA strand (43–45), rNMPs accumulation in the genome has deleterious consequences, leading to mutagenesis and replication stress (11,12,16,25). Despite the increased interest in this research area, a mechanistic connection between RNase H2 functions and AGS pathogenesis is still missing.

Knock-in mice carrying the most common AGS mutation in RNase H2 do not display any obvious phenotype, possibly due to differences in the pathways between humans and mice (11). On the other hand, RNase H2 knock-out mice or mice with almost completely abolished RNase H2 activity die early during embryogenesis or perinatally (11,12). Thus, additional cellular mammalian models are necessary to gain insights on the molecular mechanisms linking RNase H2 defects to AGS pathogenesis.

To overcome the drastic effects caused by total loss of RNase H2 activity and differences linked to the employment of murine models, we used human cells where RNase H2 expression was modulated by RNA interference. We found that, shortly after infection with lentiviral vectors carrying specific shRNA sequences, HeLa RNase H2-silenced cells showed cell cycle defects leading to accumulation of cells in S and G2/M phases. Such defects are not cell line specific since we obtained similar results with MRC5VI cell lines. Moreover, the cell cycle progression defects correlate with the activation of the PRR pathways and increased sensitivity to replication stress-inducing agents, similarly to what we previously observed in budding yeast (25). When RNase H2 activity is defective, chromosomes accumulate rNMPs that, when embedded in the template DNA strand, create an obstacle to replication fork progression. In this situation, cells need PRR activity to complete chromosomal DNA replication, as suggested by the chronic ubiquitylation of PCNA.

These findings prompted us to ask whether human cells obtained from AGS patients were also defective in ribonucleotide excision repair, were affected in normal DNA replication and were exhibiting a chronically active PRR pathway, necessary for completing chromosomal DNA replication in the presence of genomic rNMPs. We thus obtained lymphoblastoid cells carrying mutations in either the A or the B subunits of the RNase H2 enzyme. The AGS mutations we tested reduced the stability of the RNase H2 complex, in most cases leading to a detectable accumulation of unrepaired rNMPs within genomic DNA. We report, indeed, that AGS2 and AGS4 cells accumulate high levels of rNMPs that remain embedded in genomic DNA and exhibit constitutive ubiquitylation of PCNA, indicative of an active post-replication repair process linked to increased replication stress. Interestingly, cells carrying the RNase H2A-G37S mutation, exhibit a stronger accumulation of mono-ubiquitylated PCNA, correlating the level of PCNA modification to the severity of the mutation.

The low sensitivity of the assays represents a limit for the analysis of milder mutations. For this purpose, it will be necessary to improve the assay itself and/or perform further analyses under different stress conditions. Indeed, the AGS phenotype may remain quiescent for a long time and the pathological conditions may arise only after some still unknown stress stimuli (30).

Overall, we report that RNase H2 silencing in human cell lines or RNase H2 mutations in AGS patients-derived cell lines increased genome instability and activate DDR markers, likely as consequence of replication stress, in agreement with some observations in mouse cells (11,12). We also observed that RNase H2 silencing affect the whole complex stability in agreement with what recently reported *in vivo* (46). In fact, in a study of the spatiotemporal dynamics of the whole complex, it was found that AGS-associated mutations alter complex formation, efficiency of recruitment and exchange kinetics at sites of DNA replication and laser-induced DNA damage. Interestingly, this study showed that RNase H2 recruitment to DNA depends upon its catalytic activity but is also aided by its interaction with PCNA.

Our data indicate that PCNA ubiquitylation is directly linked to defects in RNase H2 as seen in AGS patients, suggesting that such PCNA modification is relevant to tolerate unrepaired genomic rNMPs, consistently with what observed in the yeast model system (25).

Altogether, we hypothesize that the DDR *per se* may, at least partially, account for the clinical symptoms related to RNase H2 mutations in AGS patients, in agreement with growing evidence linking the DDR to the innate immune response. Activation of the DDR was reported to induce cell surface expression of ligands for NKG2D, the stimulatory receptor expressed by natural killer and by T-cell sub-populations, and more recent data showed that IFN is produced in response to DNA damage (47–49). It is likely that other molecular pathways are also linked to RNase H2-dependent AGS pathogenesis. Among them a possible role of RNase H2 in retroelement metabolism must be considered. Indeed, the TREX1 and SAMHD1 genes which are mutated in a subset of AGS patients (2,3) are involved in HIV infection and/or endogenous retroelements metabolism (50–54). Moreover, RNase H2 was identified in a high-throughput screening as a factor that facilitate HIV replication likely by removing ribonucleotides incorporated by errors of HIV reverse transcriptase (21,55).

In summary, we provide evidence that the reduction in RNase H2 activity found in cells from AGS patients is responsible for the accumulation of rNMPs in genomic DNA, thus inducing replication stress that, in turn, promotes genome instability and triggers the DDR. Although other pathways likely account for the AGS symptoms, it is possible that the increase in IFN levels, a signature of the AGS disorder, is induced by the DDR itself. If this were the case it would be interesting to see whether DDR inhibition would reduce IFN level and attenuate the autoimmune response.

## MATERIALS AND METHODS

### Antibodies and chemicals

The following antibodies were used: anti-RNase H2C (ProteinTech, 1 : 1000), anti-RNase H2A (Abcam, 1 : 1000), anti-Actin (Santa Cruz Biotechnology, 1 : 10 000); anti-RNase H2B (produced in this work, 1 : 500); anti-Caspase3 (Cell Signaling, 1 : 1000); anti-PCNA (PC-10, 1 : 200) was kindly provided by S. Sabbioneda; anti-BrdU FITC-conjugated (BD Biosciences, 1 : 50), anti-Chk1 (Cell Signaling, 1 : 200); anti-Chk2 (Cell Signaling, 1 : 1000); anti-P-Chk1-Ser317 (Cell Signaling, 1 : 1000); anti-P-Chk2-Thr68 (Cell Signaling, 1 : 500); anti p53 (DO1, GeneSpin, 1 : 1000); anti-P-p53-Ser15 (Cell Signaling, 1 : 1000); anti-53BP1 (Cell Signaling, 1 : 150 for immunofluorescence; 1 : 1000 for western blot); anti-Vinculin (Sigma, 1 : 40 000); anti-Vimentin (Cell Signaling, 1:10 000); anti-Tubulin (Sigma, 1 : 1000). Secondary antibodies were goat anti-mouse or goat anti-rabbit conjugated to HRP (western blot) or to Alexa Fluor 488 or Alexa Fluor 594 (immunoﬂuorescence). Hydroxyurea was used at a final concentration of 0.1 mm. Puromycin was used to a final concentration of 1 μm.

### Cell culture

HeLa, MRC5VI and HEK293T cells were cultured in DMEM containing 10% FBS, penicillin, streptomycin and l-glutamine and kept at 37°C in a humidified atmosphere with 5% CO_2_. EBV-immortalized lymphoblastoid cells derived from patients and control cells were cultured in RPMI containing 20% FBS, penicillin, streptomycin and l-glutamine and kept at 37°C in a humidified atmosphere with 5% CO_2_.

### Lentiviral vectors production

shRNA sequences targeting subunits A or B of human RNase H2 were cloned using *Eco*RI and *Age*I in pLKO.1-TCR cloning vector (Addgene). As control SCRAMBLE shRNA cloned in pLKO.1 vectors was used (Addgene). Lentiviral vectors were produced by transient co-transfection of pLKO and packaging plasmids psPAX2 (Addgene) and pMD2.G (Addgene) into HEK293T. Virus was harvested at 48 posttransfection and infections were carried out in the presence of 10 μg/ml of polybrene. Following transduction, cells were selected with 1 μg/ml puromycin.

### shRNA sequences

Sense sequences used to silence RNase H2A or RNase H2B are: RNase H2A: 5′-GAAATGGCAGTTCGTGGAGAA-3′, RNase H2B.1: 5′-GCTTCTCCACTACCTCATAAA-3′, and RNase H2B.2: 5′-ATCAAACTGTGGCAGCATTAA-3′.

### Western blotting

Cells were lysed in 1% sodium dodecyl sulfate (SDS) sample buffer (62.5 mm Tris–HCl, pH 6.8, 2% SDS, 10% glycerol, 50 mm DTT, 0.01% bromophenol blue), sonicated 10 s and heated at 95°C for 5 min; equal amounts of total protein extracts were analyzed by SDS–polyacrylamide gel electrophoresis. Protein markers from New England Biolabs, Fermentas or GeneSpin were used.

### Total RNA extraction and retrotranscription

Total RNA was isolated using High Pure RNA isolation kit (Roche) and 0.5–1 μg of RNA was retrotranscribed using M-MLV Reverse Transcriptase (RNaseH-) (Euroclone) according to manufacturer's protocol.

### Primer sequences

RNase H2A for: 5′-GGATACTGATTATGGCTCAGGCTACC-3′; RNase H2A rev: 5′-ATGTGATCTTCCTGAGTCCCTCCT-3′; RNase H2B for: 5′-GCATCTTGTTGCTGAAACTTCCTGG-3′; RNase H2B rev: 5′-TCCTTGTCAGTGGAAGCTTGGTCA-3′; GAPDH for: 5′-AAGGTCGGAGTCAACGGATTTGGT-3′; GAPDH rev: 5′-GCTCCTGGAAGATGGTGATGGGATTT-3′.

### Proliferation and cytotoxicity assays

HeLa cells were seeded in 96-well plates at a density of 3000–5000 cells per well. Viable cells were assessed by MTS assay every 24 h for 4 days at 490 nm. Leakage of LDH was measured as an index of lethal membrane injury (necrosis) using a commercial kit (Promega).

### Cell cycle and fluorescence-activated cell sorting analysis

Exponentially growing cells were pulse labelled with 100 μm BrdU for 30 min and released in BrdU-free medium for different time points, or immediately harvested by trypsinization and washed in phosphate buffered saline (PBS), fixed in 70% ice cold ethanol and either stained for anti-BrdU (AlexaFluor 488 as secondary antibody) and propidium iodide to quantitate replicating cells. Fluorescence-activated cell sorting (FACS) analysis was performed on a BD FACScan; quantification used Cell Quest software (BD Bioscience).

### Colony forming assay

One hundred HeLa cells were plated in p60 dishes in duplicate and allowed to grow for 14 days in complete medium in the presence of 0.1 mm HU or left untreated. After 14 days, cells were fixed in methanol and stained with crystal violet 1%. Colonies of >70 cells were scored under microscope.

### Immunofluorescence

Cells were plated on glass slides, washed twice in PBS and pre-extracted for 5 min at 4°C in CSK buffer (0.1% Triton X-100 10 mm PIPES, pH 6.8, 100 mm NaCl, 300 mm sucrose, 3 mm MgCl_2_, 1 mm EGTA and 1 mm EDTA). Lymphoblastoid cells were cytospun to polysine slides (Thermo Scientific) for 4 min at room temperature, 800 rcf. Cells were then fixed in PFA 4% for 15 min at RT and washed twice in PBS. Cells were permeabilized in PBS/0, 5% Triton X-100 for 5 min at 4°C and blocked in PBS/10% BSA for 1 h at RT. Anti-53BP1 antibody was diluted 1 : 100 in PBS/0.1% Tween and incubated for 3 h at RT. Secondary antibody was diluted 1 : 1000 in PBS/0, 1% Tween and incubated for 1 h at RT. Cells were rinsed in PBST 0.1 three times for 10 min and mounted using ProLong Gold (Invitrogen) containing DAPI to stain nuclei. Images were obtained using a Leica DMRA2 Microscope (with Leica FW4000 software) with a ×63 oil immersion objective.

### Foci and micronuclei counting

Cells with more than five 53BP1 foci per nucleus were counted covering randomly whole coverslip. At least 200 nuclei were scored for each sample. For micronuclei at least 500 cells per sample were counted.

### Western blot quantification

Western blot was quantified using ImageJ software. The ratio between modified protein and total protein was normalized on loading control and expressed as fold change respect to control sample.

### Ribonucleotides incorporation assay

Genomic DNA was purified using Qiagen Genomic-Tip and Qiagen Genomic DNA buffer set and was digested with 0.5 U *Escherichia coli* RNase HII (New England Biolabs) in 50 µl at 37°C for 2.5 h. After precipitation in 0.3 m sodium acetate, pH 7, and ethanol, DNA was resuspended in TE 0, 1%. Nick translation was performed using 5 U of *E. coli* DNA polymerase I (New England Biolabs), 20 µm of unlabeled dA/-T/-GTP, and 5 µC α^32^PdCTP in a final volume of 20 µl. The reaction was incubated at 16°C for 30 min. Labeled DNA was separated from unincorporated nucleotides by electrophoresis on a 1% TAE agarose gel. After drying, the radioactive gel was analyzed using a Typhoon. The relevant ^32^P-signal of each sample was normalized on total genomic DNA measured by ethidium bromide staining. The ratio between *E. coli* RNHII-treated and untreated sample was expressed as fold change respect to the control sample.

## SUPPLEMENTARY MATERIAL

Supplementary Material is available at *HMG* online.

## FUNDING

This work was supported by Fondazione Cariplo (grant number 2013-0798) to P.P., from MIUR (FIRB RBFR10S3UQ) to F.L., by AIRC and MIUR to M.M.-F. The financial support of Telethon-Italy (grant number GGP11003) is also gratefully acknowledged. S.O., C.C. and M.B. were funded by the European Union's Seventh Framework Program (FP7/2007-2013) under grant agreement number 241779. A.P.J. has been funded by the Medical Research Council, the European Research Council and the Lister Institute of Preventive Medicine. Funding to pay the Open Access publication charges for this article was provided by Fondazione Telethon.

## Supplementary Material

Supplementary Data
