# Supplementary material for: Reduction of hRNase H2 activity in Aicardi–Goutières syndrome cells leads to replication stress and genome instability
Source: Hum Mol Genet. 2014 Sep 30;24(3):649–58. doi: 10.1093/hmg/ddu485 (PMC4291245; doi:10.1093/hmg/ddu485)
Supplement: Supplementary Data [file supp_24_3_649__index.html]

Reduction of hRNase H2 activity in Aicardi–Goutières syndrome cells leads to replication stress and genome instability — Reduction of hRNase H2 activity in Aicardi–Goutières syndrome cells leads to replication stress and genome instability — Supplementary Data 

# Reduction of hRNase H2 activity in Aicardi–Goutières syndrome cells leads to replication stress and genome instability

## Supplementary Data

Supplementary Data

**Files in this Data Supplement:**

- Supplementary Data - Pdf file
