# Supplementary material for: Reduction of hRNase H2 activity in Aicardi–Goutières syndrome cells leads to replication stress and genome instability
Source: Hum Mol Genet. 2014 Sep 30;24(3):649–58. doi: 10.1093/hmg/ddu485 (PMC4291245; doi:10.1093/hmg/ddu485)
Supplement: Supplementary Data [file supp_ddu485_ddu485supp.pdf]

SUPPLEMENTARY MATERIAL

SUPPLEMENTARY METHODS

Quantitative real-time PCR. The cDNA product was diluted 1:20 in TE. PCR primers were designed to amplify 200 bp fragments (sequences are available upon request). GAPDH was used as housekeeping control. Quantitative PCR was performed using SYBR green IQ reagent (Biorad) in the iCycler IQ detection system (Biorad). Reactions were run in triplicates, and the relative sample enrichment was calculated with the following formula: $2\Delta Ctx - 2\Delta Ctb$, where $\Delta Ctx = Ct\ input - Ct\ sample$ and $\Delta Ctb = Ct\ input - Ct\ control\ Ab$.

LEGENDS TO SUPPLEMENTARY FIGURES

Supplementary Figure 1: antibody against recombinant human RNase H2B (N-terminal 6x His tag) specifically detects RNase H2B by immunoblotting lysates from control HeLa cells (shSCR) or HeLa cells depleted of (shRNH2B) or over-expressing (PGK-RNH2B) human RNase H2B. Actin was used as loading control.

Supplementary Figure 2: MTS assay was performed in triplicate to monitor cell proliferation. MRC5VI cells were plated 7 days after infection and analyzed every 48 hours. Mean +/- s.d..

Supplementary Figure 3: The efficiency of silencing of two different shRNA sequences targeting subunit B of RNase H2 was evaluated. Expression levels of RNase H2B were

measured by quantitative RT–PCR (qRT–PCR). Relative expression was normalized against GAPDH. Error bars represent mean +/- s.d., n=3.

Supplementary Figure 4: Asynchronous MRC5VI cells were pulse labeled with BrdU and released in BrdU-free medium. Cell were harvested and stained with anti-BrdU and Propidium Iodide (PI) at indicated time points. R1=early S phase; R2= mid S phase; R3= late S/G2 phase (arrows indicate cells entered in new G1 phase)(A).
Sensitivity to HU was evaluated by colony forming assay. Control and silenced MRC5VI cells were seeded at low density in 60 mm dishes. 24 hours after plating medium 0.1 mM HU was added to the medium. Cells were incubated for 14 days and then fixed and stained with crystal violet. Only colonies containing more than 70 cells were scored. The histograms report the percentage of surviving colonies respect to the untreated sample. Two independent experiments were performed in triplicate. Mean +/- s.e.m. (B).

Supplementary Figure 5: representative images of anti-53BP1 immunostaining on control and silenced HeLa cells. Nuclei were stained using DAPI. (Scale bar: 25 μm, Magnification 63x) (A). Expression level of 53BP1 in control and RNase H2-silenced HeLa cells was evaluated by western blot. Vinculin was used as loading control (B).

Supplementary Figure 6: representative images and magnification of micronuclei (indicated with white arrows) in control and RNase H2-silenced HeLa cells. Nuclei were stained using DAPI. (Scale bar: 25 μm, Magnification 63x).

Supplementary Figure 7: Expression level of 53BP1 in control and AGS-mutated lymphoblastoid cells was evaluated by western blot. Tubulin was used as loading control.

Supplementary Figure 1 Pizzi et al

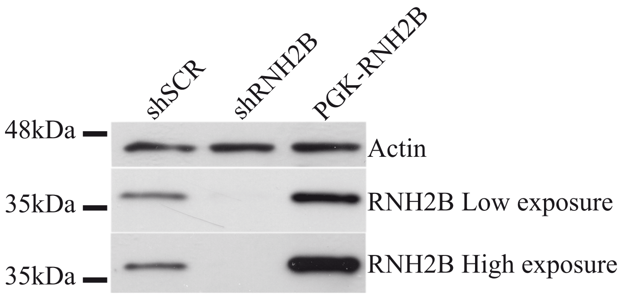

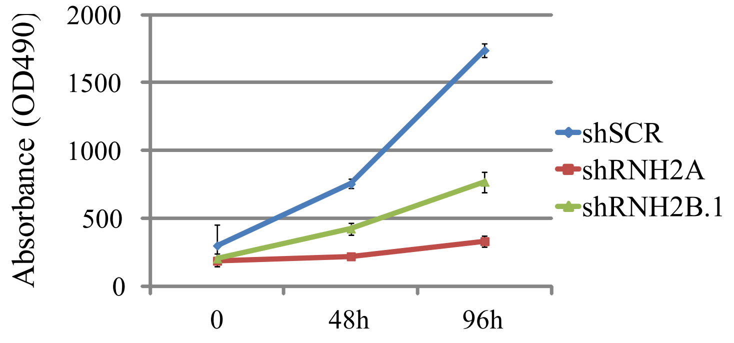
Absorbance (OD490)
2000
1500
1000
500
0
0
48h
96h
shSCR
shRNH2A
shRNH2B.1

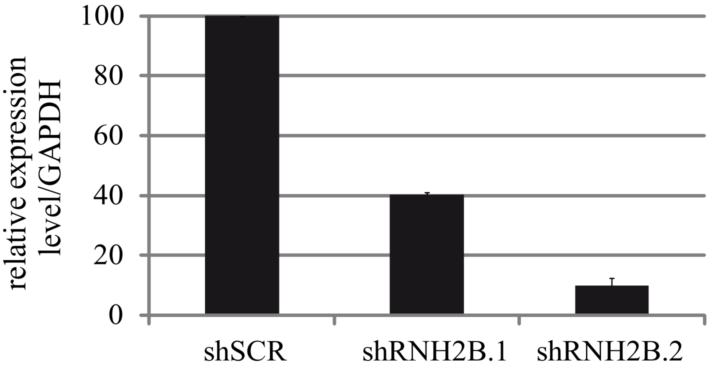
100
80
60
40
20
0
relative expression level/GAPDH
shSCR
shRNH2B.1
shRNH2B.2

A

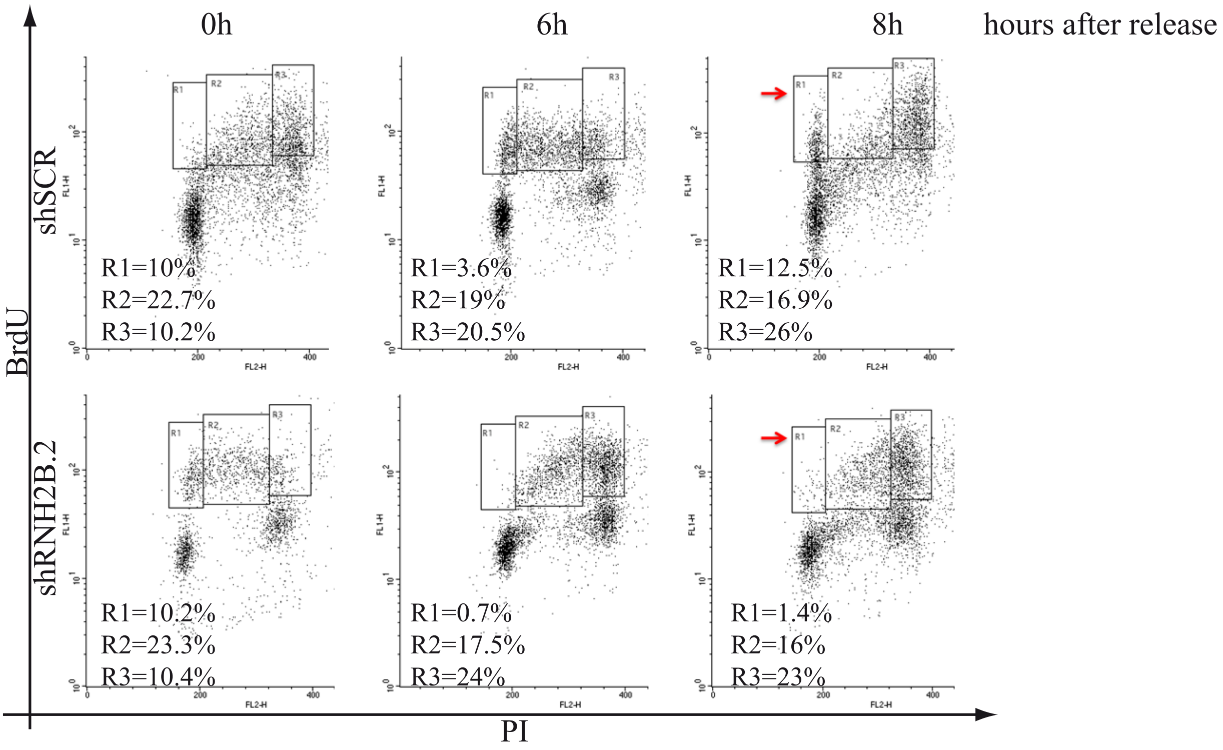
0h
6h
8h
hours after release
shSCR
shRNH2B.2
BrdU
PI
R1=10%
R2=22.7%
R3=10.2%
R1=3.6%
R2=19%
R3=20.5%
R1=12.5%
R2=16.9%
R3=26%
R1=10.2%
R2=23.3%
R3=10.4%
R1=0.7%
R2=17.5%
R3=24%
R1=1.4%
R2=16%
R3=23%

B

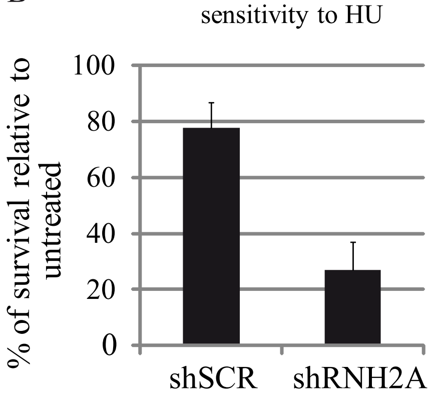
sensitivity to HU
% of survival relative to untreated
100
80
60
40
20
0
shSCR
shRNH2A

A

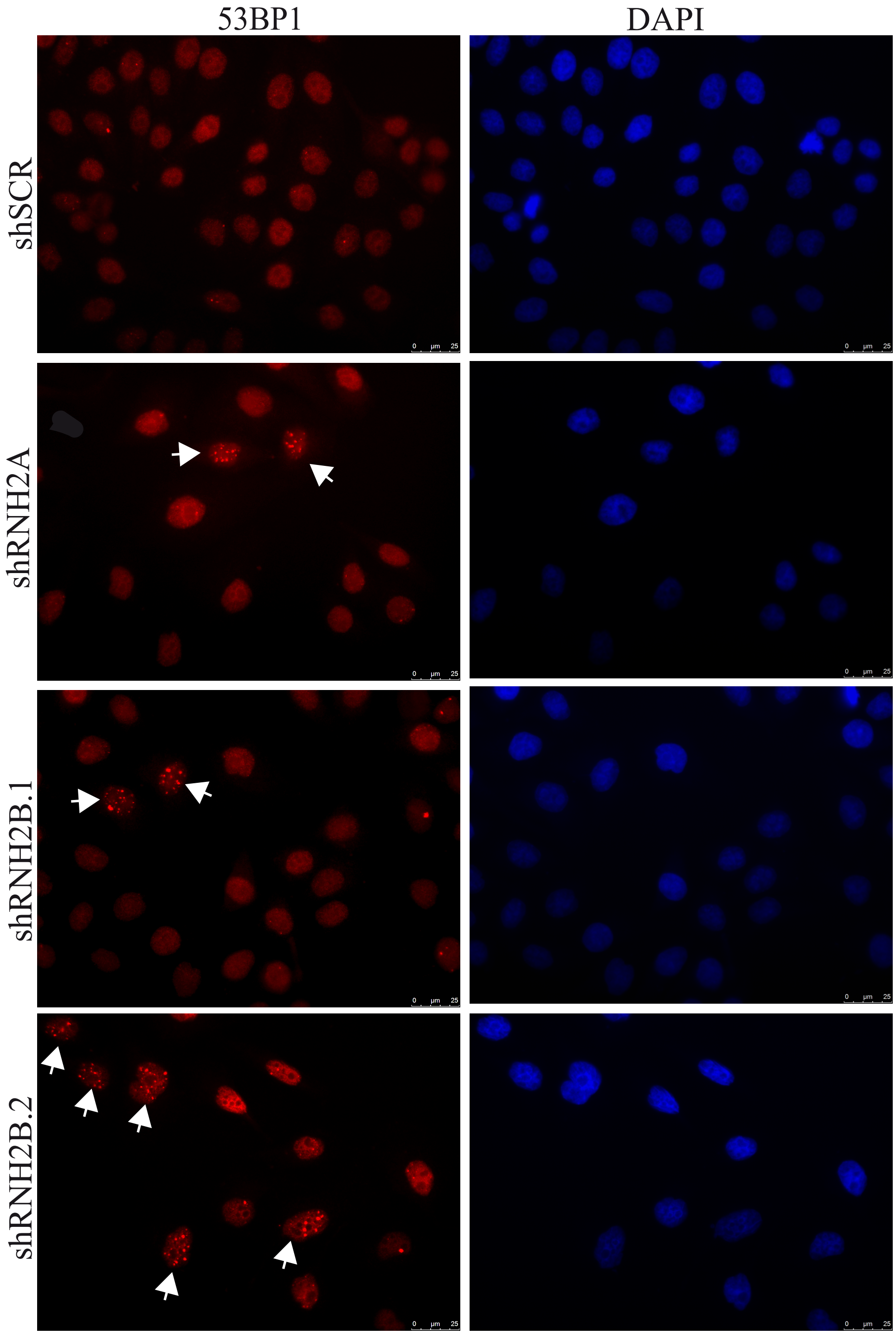
53BP1
DAPI
shSCR
shRNH2A
shRNH2B.1
shRNH2B.2

B

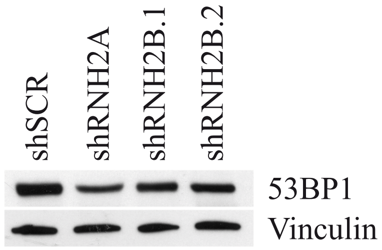
shSCR
shRNH2A
shRNH2B.1
shRNH2B.2
53BP1
Vinculin

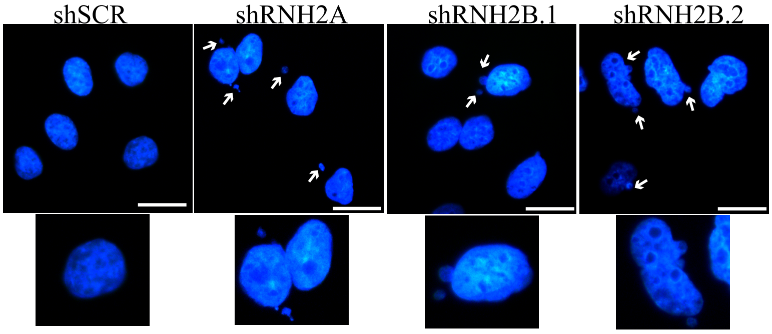
shSCR
shRNH2A
shRNH2B.1
shRNH2B.2

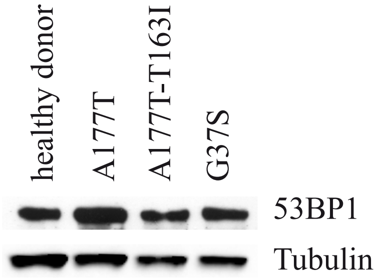
healthy donor
A177T
A177T-T163I
G37S
53BP1
Tubulin
